# Relationships between Grey Matter Volume in the Bilateral Superior Frontal Gyrus and Reactive Aggression Varied by Level of Traditional Masculinity

**DOI:** 10.3390/brainsci14060605

**Published:** 2024-06-15

**Authors:** Weijun Liu, Cody Ding, Ziang Li, Hong Chen

**Affiliations:** 1 Faculty of Psychology, Southwest University, Chongqing 400715, China; liuweijunpsy@163.com (W.L.); liziang20061122@163.com (Z.L.); 2 Key Laboratory of Cognition and Personality, Ministry of Education, Southwest University, Chongqing 400715, China; 3 Research Center of Psychology and Social Development, Southwest University, Chongqing 400715, China; 4 Department of Education Sciences & Professional Programs, University of Missouri-St. Louis, St. Louis, MO 63121-4400, USA; dingc@umsl.edu

**Keywords:** traditional masculinity, reactive aggression, proactive aggression, superior frontal gyrus, grey matter volume

## Abstract

Although previous behavioral studies have associated reactive aggression (RA) and proactive aggression (PA) with traditional masculinity, further investigation is needed into the traditional masculinity-linked neuroanatomical characteristics of RA and PA. This study analyzed the traditional masculinity-by-aggression interaction in 705 participants (355 men) by measuring grey matter volume (GMV). We have expanded on previous studies and found that traditional masculinity was not associated with RA and PA when not controlled for traditional femininity. However, the association appeared when controlling for it. Furthermore, we found significant traditional masculinity-by-RA interactions on the GMV in the bilateral superior frontal gyrus, a region known to be involved in cognitive control. When traditional masculinity scores were 1 standard deviation above the mean, there was a positive correlation between RA and the GMV in the bilateral superior frontal gyrus. Conversely, when traditional masculinity scores were 1 standard deviation below the mean, there was a negative correlation between RA and the GMV in the region. However, no traditional masculinity-linked neuroanatomical characteristics of PA were found. The results indicated that individuals with high/low traditional masculinity perceived RA as a different outcome (gain or loss) of self-control. The results supported an opportunity to develop prevention or intervention strategies for RA.

## 1. Introduction

Human aggression can be classified as reactive aggression (RA) and proactive aggression (PA) based on the motivation behind aggressive behavior [1]. RA is often defined as aggression driven by negative emotions such as anger and is enacted impulsively [2]. PA is goal-directed and characterized by low levels of emotional reactivity [3]. It is closely associated with callous–unemotional traits [4,5]. These two subtypes of aggression are more complementary than antagonistic [1], and both subtypes of aggression are detrimental to individuals and society. For instance, negative reactivity primarily fueled 82.1% of homicides perpetrated by non-psychopathic individuals [6,7]. Additionally, the number of intentional firearm deaths increased across all age groups in the United States between 2017 and 2020 [8]. In this context, we need to gain a deeper understanding of the neurobiological characteristics that underlie RA and PA because the more we know about the characteristics that underlie aggression, the more opportunities we will have to develop effective ways to prevent it [1].

Individuals gain knowledge through socialization from their parents, peers, and social media about societal expectations of gender-related attitudes and behaviors, which are typically defined as personal attributes considered desirable in a man and a woman in a given society, that is, masculinity and femininity [9,10,11]. There are various facets to masculinity and femininity, with traditional masculinity (abbreviated as masculinity) being perceived as more competitive and aggressive [12,13]. For example, Parrott and Zeichner suggested that individuals who exhibited highly hypermasculine traits displayed greater aggression on aggressive tasks and reported more frequent aggressive behavior [14]. Malonda’s investigation found a positive association between masculinity and RA and PA in 390 Spanish adolescents [15]. In summary, it is likely that aggression is higher in individuals with high masculinity scores than in those with low masculinity scores.

In contrast, traditional femininity (abbreviated as femininity) was associated more with expressiveness, such as sensitivity to the needs of others, understanding, caring, gentleness, and submissiveness [12,13]. Some studies have demonstrated that femininity is negatively associated with direct aggression [16], verbal aggression [17], and RA and PA [15,18]. Notably, the fact that masculinity and femininity affect aggression in opposite directions does not mean that these two traits are mutually opposite or exclusive. Individuals can simultaneously exhibit high levels of both masculinity and femininity or low levels of both [19]. However, the previous studies on the effects of masculinity on RA and PA did not control for the effects of femininity, which may impact the prediction of masculinity for RA and PA. Thus, we controlled for femininity when examining the relationship between masculinity and RA/PA.

Furthermore, although prior studies have examined the relationship between masculinity and aggression, the masculinity-linked neuroanatomical characteristics of RA and PA have not been considered. In other words, it is not clear whether and how the neuroanatomical changes related to aggression may be influenced by specific brain regions, such as those associated with masculinity (e.g., the frontal lobe) [20]. The neuroanatomical characteristics of RA and PA may differ among individuals with different levels of masculinity, as RA and PA also vary among those with varying levels of masculinity (e.g., 1 standard deviation above the mean vs. 1 standard deviation below the mean). Neuroanatomical characteristics could be used by clinicians, educators, and social workers to develop a prevention or intervention program suitable for individuals with different degrees of masculinity [1,21]. Thus, we further explored the masculinity-linked neuroanatomical characteristics of RA and PA using the voxel-based morphometry approach based on structural magnetic resonance imaging. 

Studies based on structural magnetic resonance imaging over the past decade have investigated the neuroanatomical characteristics (e.g., grey matter volume, GMV) in RA and PA [1]. In adults, self-reported RA scores were negatively associated with GMV in the left amygdala, which played a key role in the processing of emotions and threats from the environment [22]. In adolescents, there was a reduction of GMV in the insula, which was involved in emotional responses and cognitive processes [23]. In individuals with intermittent explosive disorder, a study reported reduced GMV in various frontal lobe regions, which was involved in cognitive control (e.g., ventromedial prefrontal cortex or orbitofrontal cortex) [24]. Similarly, higher RA was found to correlate with smaller volumes of the right middle frontal cortex [25]. In addition, few studies have examined the structural characteristics of PA, particularly in GMV. In a study of self-reported PA scores, volumes of the amygdala, posterior cingulate cortex, and lateral and medial frontal cortex were negatively related to PA in conduct disorder and oppositional defiant disorder among normative adolescents and adults [22,23,25,26]. Another study found a positive association between increased PA and the right amygdala volume and left anterior cingulate cortex [27]. Based on these previous studies of these two subtypes of aggression, the masculinity-linked neuroanatomical characteristics of RA and PA were expected to more likely encompass brain regions linked to emotional regulation (e.g., amygdala) and executive control (e.g., the frontal lobe). 

Thus, we hypothesized that masculinity was a positive predictor of RA and PA and would be strengthened by controlling for femininity (Hypothesis 1). Furthermore, we explored the masculinity-linked neuroanatomical characteristics (i.e., GMV) of RA and PA via voxel-based morphometry. We hypothesized that the neuroanatomical characteristics would be present in the amygdala or frontal lobe (Hypothesis 2).

## 2. Materials and Methods

### 2.1. Participants

The data for the study were extracted from the Behavioral Brain Research Project on Chinese Personality (BBP) [28], without any overlap with previously published BBP data. A total of 906 participants took part in this study, and, after excluding those with incomplete data, the current study was based on data from 705 subjects (350 women, *M* = 19.23 ± 1.07 years, age range 17.27 to 25.88 years; 355 men, *M* = 19.05 ± 0.89 years, age range 17.07 to 23.15 years). The Southwest University Ethics Committee for Scientific Research approved the study. Written informed consent was obtained from all participants and their parents/guardians for minors. All procedures adhered to the World Medical Association Code of Ethics (Declaration of Helsinki). 

### 2.2. Measures

To assess masculinity and femininity, participants completed the Bem Sex Role Inventory, a well-established measure comprising 20 items each related to femininity and masculinity. Using a 7-point scale ranging from 1 (*never or almost never true*) to 7 (*always or almost always true*), participants indicated the extent to which each item described their psychological traits. This instrument has been extensively utilized in Chinese research contexts and provides a reliable means of evaluating gender-related traits [29,30]. Although the Bem Sex Role Inventory was an older questionnaire, we opted to use it for two reasons. First, the inventory focused on measuring traditional masculinity and femininity, which were more closely associated with aggression [31]. Second, some research suggested that men and women still exhibited the same gender stereotypes as in the past, although the connotations of masculinity and femininity have evolved over time as society has developed [32]. In our study, the Cronbach’s alpha of the femininity and masculinity subscale was 0.83 and 0.87, respectively. 

RA and PA were assessed using the Reactive and Proactive Aggression Questionnaire [33]. This questionnaire consisted of 23 items (11 items assessing RA and 12 items assessing PA) scored on a 3-point scale from 0 (*never*) to 2 (*often*), with high scores indicating high aggression. This scale has undergone validation and has been extensively utilized in appraising RA and PA based on Chinese individuals [4,23,34]. In this study, Cronbach’s alpha for RA and PA was 0.85 and 0.84, respectively.

### 2.3. MRI Data Acquisition and Processing

Structural scanning was performed using a 3.0-T Siemens Trio MRI scanner (Siemens Medical, Erlangen, Germany). MRI images were obtained using a magnetization-prepared rapid gradient-echo T1-weighted sequence. Key imaging parameters included a repetition time of 2530 ms, echo time of 2.98 ms, inversion time of 900 ms, and flip angle of 7°, with a resolution matrix set at 256 × 256 [35]. The acquisition encompassed 176 contiguous sagittal slices with a 1.0 mm slab thickness, ensuring comprehensive coverage of the entire brain with a voxel size of 0.5 × 0.5 × 1 mm^3^. Ethical guidelines set by the Research Project Ethical Review Committee were strictly adhered to throughout the scanning process [28].

Subsequent processing of the structural MRI images was conducted using the widely employed Statistical Parametric Mapping software package (Version 12.0) [36,37]. Initially, all images underwent meticulous inspection within SPM12 to identify and address any potential artifacts or gross anatomical irregularities, enhancing the quality of subsequent analyses. Following this quality assurance step, the anatomical images were subjected to segmentation into white matter, grey matter (GM), and cerebrospinal fluid compartments utilizing the new segmentation routine within SPM12 [38]. Further processing involved registration, normalization, and modulation procedures using the DARTEL method [39], a robust technique well established in the field of Voxel-based morphometry studies [40,41]. This involved resampling GM images to a uniform voxel size of 1.5 mm × 1.5 mm × 1.5 mm, normalization to the Montreal Neurological Institute space, and smoothing with a 6 mm full width at half Maximum Gaussian kernel to enhance statistical power. Importantly, modulation was applied to preserve local GM volumes, ensuring accurate representation of structural differences across subjects. 

### 2.4. Statistical Analysis 

In addition to descriptive statistics analysis, we first performed hierarchical regression analysis to examine the relationship between masculinity and RA/PA using IBM SPSS version 25. In hierarchical regression analysis, we first entered masculinity to examine its predictive effect on aggression (Models 1 and 3). Then, we entered both masculinity and femininity to examine the predictive effect of masculinity on aggression when controlling for femininity (Models 2 and 4). We compared the predictive value of masculinity on aggression (i.e., beta) while controlling for femininity and without controlling for femininity. 

Second, to detect the masculinity-linked neuroanatomical characteristics of RA and PA (i.e., the relationship between RA/PA and GMV differs according to the degree of masculinity), a voxel-wise condition-by-covariate (i.e., masculinity-by-aggression) interaction analysis was performed using the SPM12. This analytical approach was used in previous studies that focused on sex-linked neural characteristics of the dependent variables [42,43]. Although these previous studies have used categorical variables (i.e., sex), our study differs by using continuous variables (i.e., masculinity). In this study, a high level of masculinity/aggression refers to scores one standard deviation above the mean masculinity/aggression scores (z score), while a low level of masculinity/aggression refers to scores one standard deviation below the mean masculinity/aggression scores (z score); this analytical approach was conducted using the PROCESS developed by Hayes [44].

In this whole brain analysis, sex, age, femininity, and total intracranial volume (TIV) were entered as covariates for control purposes. Significant areas were selected as regions of interest (ROIs) to analyze the effect of the masculinity-by-aggression interaction using Model 1 of SPSS PROCESS. The REX toolbox (http://gablab.mit.edu/downloads/rex.m, accessed on 6 May 2024) was used to obtain GMV means. 

Third, to test the specificity of the relationship, we performed whole brain correlation analyses between RA/PA and region GMV, with sex, age, masculinity, femininity, and TIV as null covariates or only controlling for sex, age, and TIV, without considering femininity and masculinity as covariates. In all analyses, an absolute threshold masking of 0.2 was applied to these analyses to exclude edge effects between white and grey matter [45]. The displayed and corrected results were completed using the DPABI software toolbox (version 6.0) [46] in the MATLAB platform. The results were corrected using the Gaussian random field (GRF) program for multiple comparisons (threshold: cluster *p* < 0.05 and voxel level *p* < 0.001) [47,48]. 

## 3. Results

### 3.1. Descriptive Statistics

Table 1 displays all variables’ means, standard deviations, ranges, and correlations. Masculinity was not significantly associated with RA and PA, whereas femininity was negatively associated with RA and PA. 

### 3.2. Behavioral Data Analysis

Table 2 displays the results of the hierarchical regression analyses, which provided partial support for Hypothesis 1. Although masculinity did not significantly predict RA (Model 1, *β* = −0.03, *p* = 0.416) and PA (Model 3, *β* = 0.05, *p* = 0.177), when femininity was considered as a control variable, masculinity was significantly and positively predicted RA (*β* = 0.09, *p* = 0.034, Model 2) and PA (*β* = 0.19, *p* < 0.001, Model 4). Moreover, no major multicollinearity was observed in models 2 and 4 (variance inflation factor = 1.41), which implied that the parameter estimates were reliable and stable while incorporating femininity as a control variable in the ensuing masculinity-by-aggression interaction analyses.

### 3.3. Brain Imaging Data Analysis

**Figure 1:**
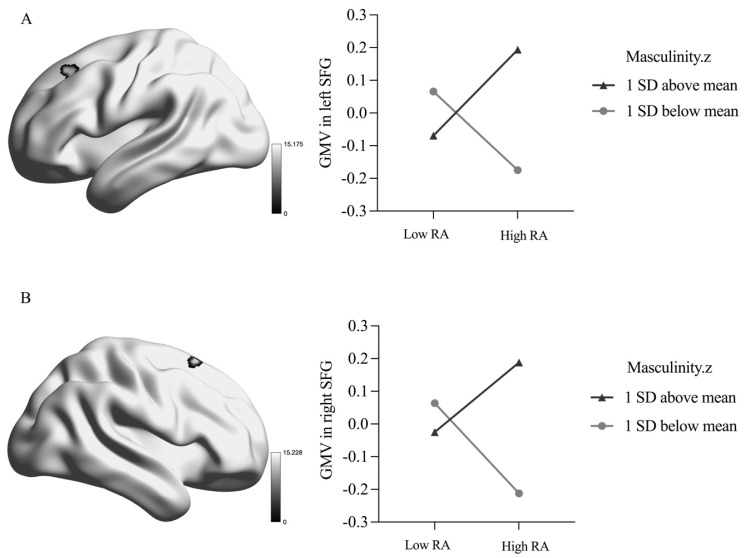
Interaction between masculinity and reactive aggression in grey matter volume. RA = reactive aggression; STG = superior frontal gyrus. (**A**) Interaction between masculinity and reactive aggression in left STG; (**B**) Interaction between masculinity and reactive aggression in right STG. Z = z-score.

There was a significant masculinity-by-RA interaction on the GMV in the bilateral superior frontal gyrus (SFG), as presented in Table 3 and Figure 1. Specifically, when masculinity was scored 1 SD above the mean, RA was positively related to the bilateral SFG GMV (left SFG: *β* = 0.13, *p* = 0.010; right SFG: *β* = 0.11, *p* = 0.038), whereas the reverse pattern was observed when masculinity was scored 1 SD below the mean (left SFG: *β* = −0.12, *p* = 0.030; right SFG: *β* = −0.14, *p* = 0.013). 

In addition, to confirm that there were indeed the neuroanatomical characteristics for RA linked to masculinity rather than neuroanatomical characteristics for RA, we conducted a whole brain correlation analysis between RA and region GMV while accounting for sex, age, TIV, femininity, and masculinity as non-interest covariates. The findings demonstrated a negative association between RA and the GMV in the straight gyrus (peak coordinate x = 0, y = 34.5, z = −27; cluster size = 254; *t* = −3.82). 

Furthermore, the straight gyrus GMV (peak coordinate x = −1.5, y = 34.5, z = −25.5; Cluster size = 400; *t* = −3.94) displayed a negative correlation with RA when only controlling for sex, age, and TIV (i.e., without considering femininity and masculinity as covariates). This result aligned with previous studies documenting similar brain regions [49,50]. Overall, these results suggested masculinity-specific correlations between bilateral SFG GMVs and RA. Yet, no masculinity-linked neuroanatomical characteristics of PA or neuroanatomical characteristics of PA were found.

## 4. Discussion

### 4.1. The Relationship between Masculinity and RA/PA

We found that RA/PA was not significantly correlated with masculinity in Models 1 and 3, which was inconsistent with Malondas’ study, showing that masculinity positively and significantly predicted RA and PA [15]. However, the regression coefficients became significant and positive after controlling for femininity in our study. One reason might be that the number of individuals with high (or low) masculinity and high (or low) femininity has increased in China compared to decades ago [51]. On the one hand, masculinity and femininity were considered risk and protective factors, respectively [31]. On the other hand, in this study, the level of masculinity (vs. femininity, 4.50 vs. 4.76, *t* = −9.75, *p* < 0.001) among Chinese university students was low, which was consistent with the findings of other studies based on the Chinese samples [51]. Accordingly, masculinity’s prediction of aggression may be suppressed by femininity in this sample. This result highlighted the importance of controlling for femininity when examining the relationships between masculinity and RA. For this reason, we also included femininity as a control variable in the subsequent interaction analysis. 

### 4.2. Masculinity and RA Interacting on Bilateral SFG

Our study revealed that the association between RA and GMV in the brain was influenced by the level of masculinity (1 SD above mean vs. 1 SD below mean). On the one hand, when masculinity was scored 1 SD below the mean, there was a negative correlation between RA and bilateral SFG GMV. This finding was consistent with previous studies suggesting the involvement of the SFG region in aggressive behavior. For example, individuals with antisocial behavior had smaller GMV in the left SFG [52], and individuals with conduct problems also had reduced GMV in the region [53]. The SFG function has been shown to play a key role in the control of impulsive responses [54]. Low masculinity meant being less likely to harm others when provoked, which was related to the low competitiveness and risk-taking that characterized socially prescribed low masculinity [51]. Individuals with low masculinity were more likely to view aggression as a loss of self-control (i.e., the aim of cognitive control was more likely to be the reduction of aggressive behavior) [55]. This corresponded to their showing a negative correlation between RA and bilateral SFG GMV. Namely, individuals are more inclined to experience high risk of RA only when their cognitive control is reduced, a result in line with prior research that found reductions in the GMV of frontolimbic structures in individuals with intermittent explosive disorder [24]. 

On the other hand, when masculinity was scored 1 SD above the mean, there was a significant positive relationship between RA and bilateral SFG GMV. The SFG was flexible in encoding certain attributes according to current goals [56] and represented the brain functional characteristics underlying aggression motivation and the ability for cognitive control [26]. Individuals with high levels of masculinity exhibited more competitive and adventurous characteristics [57]. They may be motivated to engage in more highly reactive aggressive behavior when provoked. This phenomenon was related to society’s encouragement of men to be highly competitive [51]. That is to say, individuals with high levels of masculinity were more likely to view aggression as a way of gaining self-control. This corresponded to their positive correlation between RA and bilateral SFG GMV. Functional MRI evidence also supports the suggestion that individuals with disruptive behavior disorders exhibit high activation in the dorsal lateral prefrontal cortex, which is involved in cognitive control [58]. Future studies can explore the relationship between function and structure since the enlargement of structure does not necessarily correlate with the enhancement of function.

In summary, our study showed that high RA in individuals with high masculinity was associated with larger bilateral SFG GMV, which may facilitate goal attainment, whereas high RA in individuals with low masculinity was associated with decreased bilateral SFG GMV, which reflected the decline of cognitive control. The patterns reflected how individuals with high or low masculinity perceive RA as a different outcome (gain or loss) of self-control [59]. Future prevention or intervention strategies could start by reducing this cognitive belief. Moreover, these findings indicate that SFG coding can be adapted to target different behavioral goals in individuals with different levels of masculinity and that such behavioral goals and coding are influenced by socio-culturally prescribed masculinity. 

By and large, our findings have three strengths that addressed gaps in past literature. First, the prediction of aggression by masculinity was significant only when femininity was controlled for. This finding responded to the greater prominence of femininity among young adults in China and reflected the interfering role of femininity in masculinity’s prediction of aggressive behavior. Second, we identified different neuroanatomical characteristics associated with high RA across varying levels of masculinity. This finding could aid in developing more effective prevention or intervention strategies for RA in the future.

### 4.3. Limitations

This study’s results should be interpreted with several limitations in mind. First, our study only used GMV to detect the masculinity-related neuroanatomical characteristics of aggression. Future studies should use other brain structural measures (e.g., cortical thickness) or brain functional measures to extend the investigation. Second, although age was treated as a covariate of no interest, significant differences were still observed in the brains of individuals aged between 17 and 25 [60]. Moreover, our study hypothesized neuroanatomical characteristics in the amygdala. However, we found no amygdala involvement and no masculinity-linked neuroanatomical characteristics of PA and neuroanatomical characteristics of PA, likely due to our non-clinical sample of university students (mean age = 19.14 years). This sample typically exhibits lower aggression levels and is younger compared to clinical or aggressive samples that have shown amygdala involvement [22,61,62,63]. In addition, university students may still be undergoing significant neurodevelopmental changes. The plasticity in the brain during this period might obscure subtle differences in amygdala structure. The findings may not be generalized to other samples. Future research should include a broader range of samples, including individuals with varying aggression levels or using longitudinal designs to track neuroanatomical changes over time could provide more insights. Moreover, this study focused exclusively on the neural basis of aggression specific to masculinity. Future research should investigate the neural underpinnings of aggression associated with femininity, which could offer insights into aggression from a protective factor perspective. Such studies would provide a more comprehensive understanding of how gender traits influence aggression and related neuroanatomical characteristics. Last, the study was based on Chinese participants. However, masculinity and femininity are strongly influenced by culture. Given the influence of culture on brain development [64], cultural factors should be considered for generalizing our findings. 

## 5. Conclusions

Our research provides empirical evidence for the masculinity-linked neuroanatomical characteristics of RA. We have found an inverse relationship between bilateral SFG GMV and RA under low or high masculinity levels in a large sample of young adults. The relationship was influenced by whether the individuals perceived aggression as representing a loss or gain of self-control. Future studies could develop prevention or intervention procedures to decrease RA in people with different levels of masculinity.

## Figures and Tables

**Table 1:** Descriptive statistics and correlations of all variables.

Variables	Mean (SD)	Range	1	2	3	4
1. Age	19.14 (0.99)	17.06–25.88				
2. Masculinity	4.50 (0.77)	2.05–6.70	0.04			
3. Femininity	4.76 (0.69)	2.50–6.80	0.01	0.54 ***		
4. Reactive aggression	6.94 (3.92)	0–20	0.01	−0.03	−0.18 ***	
5. Proactive aggression	1.31 (2.55)	0–16	0.04	0.05	−0.15 ***	0.43 ***

Note. *** *p* < 0. 001. SD = standard deviation.

**Table 2:** Regression coefficients of reactive and proactive aggression.

		Δ*R*^2^	*F*	*β*	VIF
Dependent variable	Reactive aggression				
Model 1	Masculinity	<0.001	0.66	−0.03	1.00
Model 2	Masculinity	0.04	14.01 ***	0.09 *	1.41
	Femininity			−0.23 ***	1.41
Dependent variable	Proactive aggression				
Model 3	Masculinity	<0.005	1.82	0.05	1.00
Model 4	Masculinity	0.05	17.85 ***	0.19 ***	1.41
	Femininity			−0.25 ***	1.41

Note. * *p* < 0.05; *** *p* < 0.001. VIF = variance inflation factor. Model 1 and Model 3 demonstrated the relationship between masculinity and aggression without controlling for femininity. Model 2 and Model 4 showed that when controlling for femininity, the relationship between masculinity and aggression became more significant, without resulting in serious multicollinearity.

**Table 3:** Brain regions significant interaction effects between masculinity and reactive aggression on grey matter volume.

			Coordinates			
Anatomical Region	Hemisphere	Cluster Size	x	y	z	*F*
STG	Left	103	−19.5	22.5	48	15.18
STG	Right	72	19.5	21	61.5	15.23

Note. STG = superior frontal gyrus.

## Data Availability

Where reasonable, data are available from the corresponding author. The data are not publicly available to protect participants’ privacy.
