# Correction: Liu et al. Relationships between Grey Matter Volume in the Bilateral Superior Frontal Gyrus and Reactive Aggression Varied by Level of Traditional Masculinity. *Brain Sci.* 2024, *14*, 605

**DOI:** 10.3390/brainsci15060627

**Published:** 2025-06-11

**Authors:** Weijun Liu, Cody Ding, Ziang Li, Hong Chen

**Affiliations:** 1 Faculty of Psychology, Southwest University, Chongqing 400715, China; liuweijunpsy@163.com (W.L.); liziang20061122@163.com (Z.L.); 2 Key Laboratory of Cognition and Personality, Ministry of Education, Southwest University, Chongqing 400715, China; 3 Research Center of Psychology and Social Development, Southwest University, Chongqing 400715, China; 4 Department of Education Sciences & Professional Programs, University of Missouri-St. Louis, St. Louis, MO 63121-4400, USA; dingc@umsl.edu

## Correction to Participant Demographics

There is a typographical error in the description of participant demographics in the original publication [1], where the number of male and female participants was mistakenly reversed, and the corresponding mean age values were assigned to the incorrect gender groups. The originally published statement—“705 subjects (350 men, M = 19.23 ± 1.07 years, age range 17.27 to 25.88 years; 355 women, M = 19.05 ± 0.89 years, age range 17.07 to 23.15 years)”—should be corrected to “705 subjects (350 women, M = 19.23 ± 1.07 years, age range 17.27 to 25.88 years; 355 men, M = 19.05 ± 0.89 years, age range 17.07 to 23.15 years)”. This error appears in Section 2.1 Participants. And in the Abstract, ‘350 men’ should be corrected to ‘355 men’. The necessary corrections should be made accordingly. Importantly, this mistake is purely typographical and does not affect any data analysis or the study’s conclusions.

The authors state that the scientific conclusions are unaffected. This correction was approved by the Academic Editor. The original publication has also been updated.
